# IL-17A deletion reduces sevoflurane-induced neurocognitive impairment in neonatal mice by inhibiting NF-κB signaling pathway

**DOI:** 10.1080/21655979.2022.2090608

**Published:** 2022-06-26

**Authors:** Qi Zhang, Yanan Li, Chunping Yin, Mingyang Gao, Jiaxu Yu, Junfei Guo, Xiaohui Xian, Zhiyong Hou, Qiujun Wang

**Affiliations:** a Department of Anesthesiology, Hebei Children’s Hospital Affiliated to Hebei Medical University, Shijiazhuang, Hebei, China; b Department of Anesthesiology, The Third Hospital of Hebei Medical University, Shijiazhuang, Hebei, China; c Department of Orthopaedics, The Third Hospital of Hebei Medical University, Shijiazhuang, Hebei, China; d NHC Key Laboratory of Intelligent Orthopaedic, The Third Hospital of Hebei Medical University, Shijiazhuang, Hebei, China; e Department of Pathophysiology, Hebei Medical University, Shijiazhuang, Hebei, China; f Department of Anesthesiology, The Third Hospital of Hebei Medical University, Shijiazhuang, Hebei, China

**Keywords:** IL-17A, sevoflurane, NF-κB signaling pathway, neuroinflammation, cognitive dysfunction

## Abstract

We investigated the role of IL-17A in sevoflurane-inducedneurocognitive impairment in neonatal mice. Seventy-two wild-type (WT) and 42 IL-17A knockout (KO) neonatal mice were randomly divided into WT (*n* = 36), IL-17A^−/−^ (*n* = 6), sevoflurane (Sev, *n* = 36), and IL-17A^−/−^ + sevoflurane (IL-17A^−/−^ + Sev, *n* = 36) groups. The latter two groups were given 3% sevoflurane for 2 h per day on postnatal days (P) 6–8. Behavioral experiments were performed on P30–36. At P37, RNA sequencing and qRT-PCR of the hippocampus was performed, neurons were detected by Nissl staining, and neuropathological changes were evaluated by HE staining. NF-κB pathway-related proteins were evaluated by western blot and immunofluorescence analyses, IL-1β and IL-6 levels were assessed by ELISA. RNA sequencing identified 131 differentially expressed genes, highlighting several enriched biological processes (chemokine activity, immune response, extracellular region, extracellular space, inflammatory response) and signaling pathways (IL-17 signaling pathway, chemokine signaling pathway, cytokine–cytokine receptor interaction, ECM–receptor interaction and influenza A). Repeated sevoflurane exposures induced long-term cognitive impairment in WT mice. The cognitive impairment was comparatively less severe in IL-17A KO mice. In addition, the increased levels of NF-κB p65, iNOS, COX-2, IL-17A, IL-6 and IL-1β, reduced neuronal numbers and neuropathological changes were ameliorated in neonatal mice in the IL-17A^−/−^ + Sev group compared with neonatal mice in Sev group. IL-17A deletion protects against long-term cognitive impairment induced by repeated sevoflurane exposure in neonatal mice. The underlying mechanism may relate to inhibiting NF-κB signaling pathway as well as the reducing neuroinflammation.

## Graphical Abstract

**Figure:**
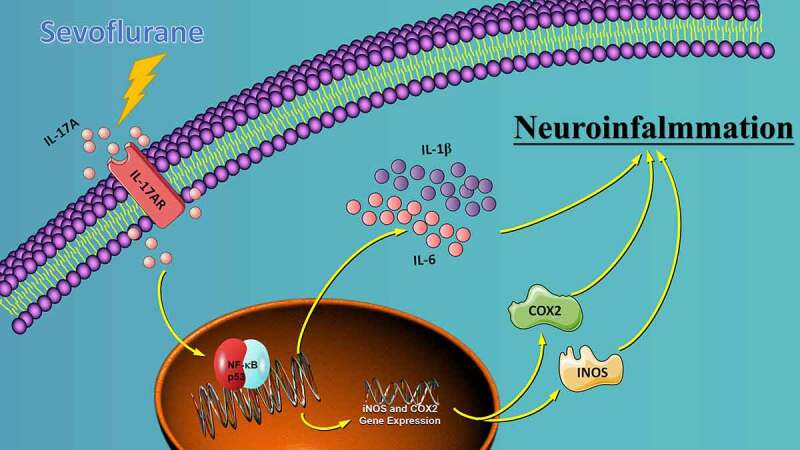

## Highlights

- IL-17A is upregulated in sevoflurane induced cognitive impairment in neonatal mice.
- IL-17A deletion alleviates sevoflurane induced cognitive impairment.
- IL-17A deletion reduces sevoflurane-induced neuroinflammation.
- IL-17A deletion inhibits activation of NF-κB signaling pathway.

## 1. Introduction

Repeated exposure to inhaled anesthetics in young children has been shown to affect long-term learning and cognitive functions. In 2016, the FDA issued a warning – for infants under 3 years of age or women in the last 3 months of pregnancy, repeated or prolonged use of general anesthesia drugs will have an adverse effect on brain development in fetuses and children. A recent retrospective study, the Mayo Anesthesia Safety in Kids (MASK) study, found that children who received repeat (≥2) and long-term (>2 h) general anesthesia before 3 years of age showed a remarkable decline in memory and fine motor abilities, problem with attention, reduced processing speed, and impaired language expression function in adolescence and adulthood [1].

Sevoflurane is the most commonly employed inhaled anesthetic in the clinic [2,3]. Animal experiments and retrospective clinical studies show that sevoflurane anesthesia of long duration or multiple exposure during development is closely related to the occurrence of cognitive impairment, which may have an adverse effect on the long-term quality of life of infants [4,5]. Therefore, studies are urgently required to investigate the mechanisms of cognitive impairment caused by multiple sevoflurane exposures to develop effective preventive measures.

Although the mechanism of neurotoxicity of sevoflurane is not completely clear, many studies have shown that the pathogenesis of sevoflurane-induced cognitive impairment may be related to neuroinflammation [6–8]. Recent studies have suggested that sevoflurane causes neuroinflammation by activating microglia [9] and peripheral immune cells, destroying the permeability of the blood–brain barrier [10], decreasing gut microbiota [11] and modulating cholinergic synaptic transmission, which can lead to cognitive impairment [12]. The hippocampus, a center for learning and memory, robustly expresses IL-1β receptor, which play key roles in learning and memory as pro-inflammatory factor receptors. However, in the pathological state, such as exposure to sevoflurane, the hippocampus is more vulnerable to the damage induced by pro-inflammatory factors, thus leading to cognitive impairment [13,14].

Interleukin 17A (IL-17A), a cytokine discovered relatively by researchers in 1993, is the first and a very important member in the IL-17 family [15,16]. Subsequent studies found that IL-17A played an important role in host defense, autoimmune diseases and tumors. Therefore, the study of IL-17A has gradually become a hot spot in medical and immunological research [17]. IL-17A has been shown to induce pro-inflammatory cytokine secretion, such as IL-1β and IL-6 [18]. IL-17A can aggravate the inflammatory response and participate in the occurrence and development of various inflammatory conditions, including cerebral ischemia-reperfusion injury, Alzheimer’s disease and rheumatoid arthritis [19]. The NF-κB signaling pathway exerts a pivotal effect in the cellular inflammatory and immune responses [20]. Perturbed regulation of the NF-κB pathway may lead to autoimmune diseases and chronic inflammation [21]. Recent studies show that NF-κB is a downstream effector of IL-17A, which plays a role in postoperative cognitive impairment after surgical anesthesia by activating the NF-κB signaling pathway of aged rats [22]. Although similar mechanisms are involved in senile postoperative cognitive impairment as well as sevoflurane neurotoxicity to the developing brain, there has been no study on the role of IL-17A in long-term cognitive impairment induced by repeated sevoflurane exposure.

Given that, we posited that there may be an intimate connection between IL-17A, the NF-κB signaling pathway, and sevoflurane neurotoxicity in neonatal rats. Thus, in this study, we investigate whether repeated sevoflurane exposure activates the NF-κ B signaling pathway by regulating the expression of IL-17A. Furthermore, we examine whether it induces the release of inflammatory factors and causes neuronal damage and long-term cognitive impairment in neonatal rats. We use the Morris water maze (MWM) test and Nissl staining to evaluate cognitive deficits and histopathological injury after repeated exposure to sevoflurane. Furthermore, to elucidate the underlying mechanism, IL-17A knockout (KO) mice were employed, combined with RNA sequencing (RNA-seq) analysis, ELISA, as well as reverse transcription-polymerase chain reaction (RT-PCR) and western blotting *in vivo*.

## 2. Materials and methods

### 2.1 Experimental animals and model preparation

A total of 72 wild-type (WT) neonatal mice (6 days of age, weighing 6–8 g, obtained from Beijing Vital River Laboratory Animal Technology Co., Ltd.) and 42 IL-17A knockout (KO) neonatal mice (6 days of age, weighing 6–8 g, purchased from Cyagen Biotechnology Co., Ltd.) were included in this study. WT neonatal mice were randomly classified into two groups: WT (*n* = 36) and WT + sevoflurane (Sev group, *n* = 36). IL-17A KO mice were assigned into two groups: IL-17A KO + sevoflurane group (IL-17A^−/−^ + Sev group, *n* = 36) and IL-17A^−/−^ group (*n* = 6). The animal use protocol was approved by the Animal Review Committee of the Third Hospital of Hebei Medical University (Code of Ethics: 2017–026-1).

**Figure 1.**
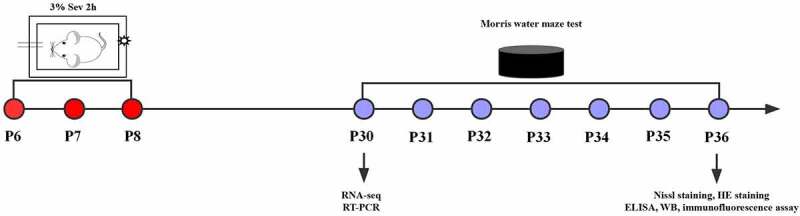
Experimental flow chart. Mice received anesthesia on postnatal days 6, 7 and 8 (P6–8) with 3% sevoflurane supplemented with 60% oxygen for 2 h daily. RNA-seq and RT-PCR were performed at P30, the Morris water maze test was conducted on P30 to P36. At P36, after the Morris water maze test, Nissl staining, HE staining, ELISA, western blot and immunofluorescence assay were performed.

To induce general anesthesia in neonatal mice, both maternal and neonatal mice were placed in an acrylic anesthesia room with two ports, one connected to a sevoflurane vaporizer (Drager, Lubeck, Germany) and the other to a multi-gas monitor (Datex-Ohmeda, Madison, WI, USA). Mice underwent anesthesia on postnatal days 6, 7 and 8 with 3% sevoflurane supplemented with 60% oxygen daily for 2 h for 3 consecutive days in the Sev and IL-17A^−/−^ + Sev groups [23]. The mice in the WT group were exposed to 30% humidified oxygen (70% N_2_) in the sevoflurane-free acrylic anesthesia room (Figure 1).

### 2.2 RNA isolation and sequencing

RNA sequencing were performed between WT and Sev group, IL-17A^−/−^ + Sev and IL-17A^−/−^ group. On P30, hippocampal tissue (6 mice from the WT group, 6 mice IL-17A^−/−^ group and 12 mice from the Sev group) were isolated on ice, followed by isolation and purification of total RNA with the use of TRIzol reagent (Invitrogen, Carlsbad, CA, USA) according to the manufacturer’s protocol. The quantification of RNA amount and purity of each sample was performed with the use of NanoDrop ND-1000 (NanoDrop, Wilmington, DE, USA). The assessment of RNA integrity was performed with an Agilent 2100, with RIN >7.0.

Purification of poly(A) RNA from total RNA (5 μg) was carried out with the use of poly-T oligo-attached magnetic beads for two rounds, followed by fragmentation of the poly(A) RNA into small pieces with the use of divalent cations under high temperature. The RNA fragments cleaved were reverse-transcribed into cDNA, for synthesis of U-labeled s-stranded DNAs using E. coli DNA polymerase I, dUTP and RNase H. The blunt ends of each strand was added with an A-base, followed by ligation to modified Illumina multiplex barcode adapters, including custom Unique Molecular Identifiers to minimize sequence-dependent bias as well as amplification noise based on Shiroguchi [24], and selection of the size was carried out using AMPureXP beads. Following the U-labeled s-stranded DNAs treatment using the heat-labile UDG enzyme, amplification of the ligated products was performed by PCR with the following conditions: initial denaturation at 95°C for 3 min, followed by denaturation at 98°C for 15 s, annealing at 60°C for 15 s, and extension at 72°C for 30 s for 8 cycles, and by a final extension at 72°C for 5 min. The average insert size for the final cDNA library was 300 (± 50) bp. Ultimately, according to the recommended protocol, paired-end sequencing was carried out using Illumina HiSeq 4000 (LC Bio, China). DEseq was employed for identification of differentially expressed genes (DEGs) and significant genes with fold change >2, and an adjusted *p*-value of <0.05 was used for analysis.

### 2.3 Functional annotation of DEGs

Gene Ontology (GO) analysis was conducted for evaluation of the functional enrichment of DEGs in biological process, cellular component, as well as molecular function. Kyoto Encyclopedia of Genes and Genomes (KEGG)-based pathway analysis was performed for the analysis of the signaling pathways enriched among the DEGs. Gene Set Enrichment Analysis (GSEA) was used for enrichment analysis of all genes, and the GSEA pathway was mapped [25,26].

### 2.4 PPI network construction and hub gene identification

The PPI networks were predicted with the STRING database (http://string‐db.org; version 11.5). The PPI networks of DEGs were constructed with a combined score >0.4, and the network was visualized with Cytoscape (version 3.8.0). CytoHubba was used to explore hub genes. The top 10 genes calculated with the highest degrees were identified as hub genes [27,28].

### 2.5 RT-PCR

RNA was extracted from the hippocampus, and synthesis of cDNA was carried out with the use of a reverse transcription kit (Transgen Biotech, Beijing, China). SYBR Green MasterMix was employed for RT-PCR in a 20 μL reaction volume with the use of the 7500 Fast Real-Time PCR System (Applied Biosystems, Bedford, MA, USA). Primer 3 version 4 (http://bioinfo.ut.ee/primer3-0.4.0/) was employed for generation of the following primers: forward primer 5ʹ-TGCTTCTGAGCCTGGTGGCTA-3ʹ; reverse primer: 5ʹ-TCAGAAATGGGGCTGGGTCT-3ʹ, with a fragment length of 495 bp. The PCR conditions were 95°C for 15 min for initial denaturation, and 35 cycles of 45 s at 95°C, 45 s at 57°C, and 45 s at 72°C (denaturation, annealing and extension, respectively). The PCR was conducted using cDNA (2 μL), nuclease-free H_2_O (19 μL), forward primer (2 μL), reverse primer (2 μL), and HotStarTaq Master Mix Kit (Qiagen, 25 μL), and the resulting products underwent electrophoresis on agarose gels containing 0.01% ethidium bromide, and images were acquired on a Protein Simple FluorChem E System (San Jose, CA, USA). Images were analyzed using Image J2x software, and the target gene expression level was expressed as a ratio of the optical density of the reference GAPDH band.

### 2.6 MWM test

At 3:00–5:00 pm on P30 to P36, the MWM test was employed for evaluation of memory and learning abilities, as previously described [23]. A round metal pool with a diameter of 150 cm and a height of 60 cm, was filled with water (1.0 cm in height) above the top of a 15-centimeter diameter platform filled with water (24–26°C). The mice facing the pool wall were slowly placed into the water from each quadrant to search for the platform. Mice were allowed to stay on the platform for 15 s after successful search. Mice were guided to the platform gently in case of failure after 60 s and allowed to stay on the platform for 15 s. At the end of the reference training (P36), after removal of the platform from the pool, mice were dropped from the opposite quadrant into water twice and allowed to explore freely for 120 s. The video tracking system was used to document the number of times the mouse crossed the hidden platform and track. At the end of each trial, the mouse was exposed to a heat lamp in a cage for 1–2 min, dried, and returned to its normal cage. JLBehv-MWM system (Shanghai Ji’Liang Software Technology Co., Ltd.) was used for the MWM test.

### 2.7 Nissl staining

At P36, after the MWM test, five mice from each group were sacrificed after deep anesthesia with sodium pentobarbital (60 mg/kg, i.p.), and the brain tissues were collected on ice and fixed in 4% paraformaldehyde. Paraffin sections were sequentially immersed in xylene and a graded alcohol series for deparaffinization, and then stained with 1% cresyl violet for 20 min at room temperature. Afterward, they were immersed and washed once in distilled water, followed by differentiation with 70% alcohol. Finally, the sections were immersed in a graded alcohol series and xylene for 2 min each. After neutral balsam mounting, the sections were observed under an optical microscope and then photographed [29].

### 2.8 Hematoxylin and eosin (HE) staining

At P36, after the MWM test, five mice from each group were sacrificed, followed by collection of brain tissues, which was then rinsed with phosphate buffer solution, and immersed in 4% paraformaldehyde for 24 h. After dehydration and embedment of the samples in paraffin wax, the paraffin-embedded 5 μm coronal brain sections containing the hippocampus were sliced and HE-stained. Optical microscopy was used for examination of the pathological changes in the hippocampus.

### 2.9 ELISA assay

Hippocampal tissues from mice in each group were isolated (*n* = 5), and 30 mg of tissue was processed into 10% hippocampal homogenate with normal saline, followed by centrifugation at 4,000 × *g* for 15 min. After collection of the supernatant, ELISA kits (Wuhan Boster Biological Technology Co., Ltd.) were employed for detection of IL-1β and IL-6 levels in the hippocampal tissue samples.

### 2.10 Western blotting

Hippocampal tissues were isolated from mice of each group (*n* = 10) and then lysed with RIPA buffer containing protein phosphatase inhibitor (Sigma-Aldrich). Total proteins were extracted and transferred onto a PVDF membrane, followed by blockage for 2 h, and incubation with primary antibodies [IL-17A antibody (ab9056), iNOS antibody (ab15323), NF-κB p65 antibody (ab207297), and COX-2 antibody (ab15191)(all 1:1,000; Abcam) and secondary antibodies. In this study, β-actin served as the internal reference protein. The ultra-sensitive chemiluminescent liquid-based FujiFilm LAS 4000 imaging analyzer (FujiFilm, Tokyo, Japan) was used for visualization, and Image J (NIH, Bethesda, MD) was used to analyze the relative intensities of individual bands.

### 2.11 Immunofluorescence assay

At P36 after the MWM test, five mice from each group were sacrificed to collect brain tissues which were then rinsed with phosphate buffer solution. For immunofluorescence staining, the hippocampus was sliced into sections (20-μm in thickness). After blockage for 2 h at room temperature with 5% bovine serum album (BSA; Beyotime, Beijing, China) containing 0.3% Triton X-100 (Sigma-Aldrich, Munich, Germany) in PBS, the samples were incubated with anti-NeuN antibody (1:200; Millipore, MAB377) and anti-NF-κB p65 antibody (1:100; Abcam, ab16502) overnight at 4°C. The sections were rinsed with PBS three times for incubation with goat anti-rabbit IgG-FITC secondary antibody (1:100; Beyotime, P0186) for 1 h at room temperature. Ultimately, staining of sections was performed with the use of 4ʹ,6-diamidino-2-phenylindole (DAPI; Beyotime, P0131) for 10 min, followed by acquisition of images on a Nikon Eclipse CI fluorescence microscope, and analysis using Image Pro Plus 6.0 software (Media Cybernetics, Inc., Rockville, MD, USA)

### 2.12 Statistical analysis

Statistical analysis was conducted with the use of SPSS version 21.0 (SPSS, Inc., Chicago, IL, USA). All data are expressed as mean ± standard deviation (SD). Concerning data with a normal distribution, differences were evaluated with the use of one-way analysis of variance (ANOVA), coupled with Tukey’s multiple comparison test. By contrast, the differences were assessed with the use of the Kruskal–Wallis test, in addition to the Dunn–Bonferroni test, with respect to a non-normal distribution. Two-way repeated-measures ANOVA as well as Tukey’s post hoc test were employed for analysis of the escape latency on training days. *P* < 0.05 was considered statistically significant.

## 3. Results

### 3.1 RNA-seq analysis identified 33 upregulated and 98 downregulated DEGs

**Figure 2.**
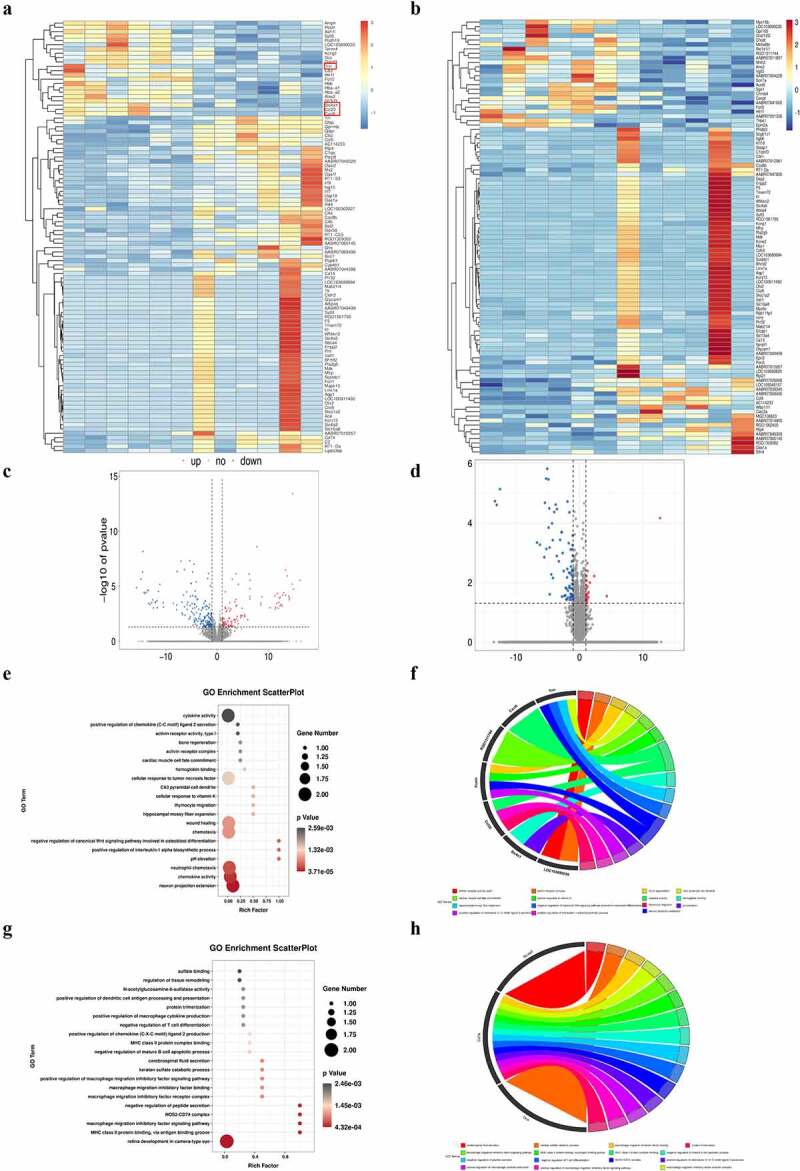
DEG identification and GO analysis of DEGs. (a) Representative heat map of differentially expressed genes in WT and WT + sevoflurane group. (b) Representative heat map of differentially expressed genes in IL-17A^−/−^ and IL-17A^−/−^ + Sev group. (c) Differential gene volcano map in WT and WT + sevoflurane group. (d) Differential gene volcano map in IL-17A^−/−^ and IL-17A^−/−^ + Sev group. (e-f) GO enrichment analysis upregulated pathways in WT and WT + sevoflurane group. (g-h) GO enrichment analysis downregulated pathways in WT and WT + sevoflurane group.

RNA-seq analysis was conducted to explore the expression of 32,623 genes in the hippocampus of the WT and Sev groups. In total, 131 DEGs were identified (Figure 2(a,c)), including 33 upregulated genes and 98 downregulated genes. These DEGs displayed differential expression profiles, showing the distinguishment between the sevoflurane group and the WT group. The top 20 upregulated and downregulated DEGs are listed in Table 1.

**Table 1.** Top 10 up-regulated and down-regulated DEGs.

Gene ID	Gene name	Transcript ID	Log_2_FC	P value
**Up-regulated gene**				
ENSRNOG00000015992	Ccl20	ENSRNOT00000021730	13.39	3.84072981942949E-10
ENSRNOG00000002843	Cxcl6	ENSRNOT00000003823	3.71	3.39523089132731E-07
ENSRNOG00000050760	LOC103690035	ENSRNOT00000073192	12.71	0.0000688090643603203
ENSRNOG00000012660	Postn	ENSRNOT00000084527; ENSRNOT00000017453	1.45	0.000815097780700335
ENSRNOG00000002548	Tnn	ENSRNOT00000003452; ENSRNOT00000076616	3.44	0.00200342823930197
ENSRNOG00000016164	Fcrl2	ENSRNOT00000021938	1.26	0.00223843358506834
ENSRNOG00000020951	Slc4a1	ENSRNOT00000092102; ENSRNOT00000028445	1.98	0.00348500772958391
ENSRNOG00000000716	Htr1f	ENSRNOT00000000907	1.29	0.00411615717014051
ENSRNOG00000005960	RGD1311744	ENSRNOT00000036054	1.11	0.00607683506368763
ENSRNOG00000001915	Chodl	ENSRNOT00000031889; ENSRNOT00000090878	2.33	0.00609892431996525
**Down-regulated gene**				
ENSRNOG00000008816	Gpnmb	ENSRNOT00000011945	−1.70	1.06602538530999E-10
ENSRNOG00000047350	Gns	ENSRNOT00000064349	−14.85	8.78140095997793E-08
ENSRNOG00000047657	C4a	ENSRNOT00000083833; ENSRNOT00000072377; ENSRNOT00000078871	−2.09	1.73016156759426E-07
ENSRNOG00000023340	Tmem72	ENSRNOT00000037397	−5.09	1.52166007039475E-06
ENSRNOG00000018735	Cd74	ENSRNOT00000025344; ENSRNOT00000025354	−1.03	2.26256481219666E-06
ENSRNOG00000039107	Mfrp	ENSRNOT00000059813; ENSRNOT00000085578	−5.27	3.25447958914502E-06
ENSRNOG00000010378	Slc4a5	ENSRNOT00000082336; ENSRNOT00000014249	−4.89	3.45959002077057E-06
ENSRNOG00000016275	Ttr	ENSRNOT00000022113	−5.03	4.79364146850667E-06
ENSRNOG00000047471	LOC103689994	ENSRNOT00000064063	−12.55	7.34042831884681E-06
ENSRNOG00000048322	LOC100911492	ENSRNOT00000074488	−13.33	0.00609892431996525

### 3.2 GO analysis and KEGG pathway analysis

**Figure 3.**
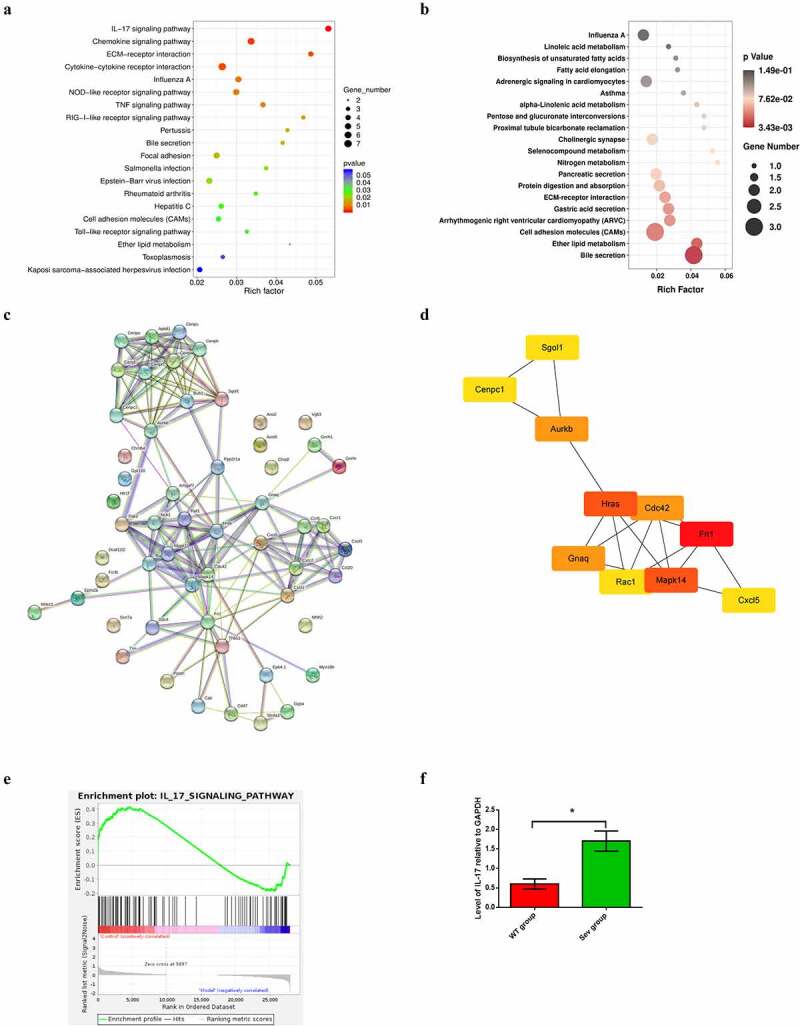
KEGG pathway analysis of DEGs, PPI network construction and hub gene identification (a) KEGG pathway enrichment scatter diagram in WT and WT + sevoflurane group. (b) KEGG pathway enrichment scatter diagram in IL-17A^−/−^ and IL-17A^−/−^ + Sev group. (c) The protein–protein interaction network of DEGs in WT and WT + sevoflurane group. (d)Ten hub genes with the highest degree in WT and WT + sevoflurane group(Red square indicates a higher degree, and yellow square indicates a lower degree). (e) GSEA gene enrichment analysis diagram. (f) Differential expression of IL-17 was confirmed by RT-PCR. Data are expressed as mean ± SD. Compared with WT group, *P < 0.05.

GO analysis suggested that the top 5 enriched GO terms among the upregulated DEGs were chemokine activity, immune response, extracellular region, extracellular space, and inflammatory response. GO pathway diagram revealed negative regulation of canonical Wnt signaling pathway involved in osteoblast differentiation, positive regulation of interleukin-1 alpha biosynthetic process, pH elevation, hippocampal mossy fiber expansion, thymocyte migaration were the top 5 enriched GO terms among the upregulated DEGs, nagetive regulation of peptide secretion, NOS2-CD74 complex, macrophage migration inhibitory factor signaling pathway, MGC class II protein binding via antigen-binding groove, cerebrospinal fluid secretion were the top 5 enriched GO terms among the downregulated DEGs(Figure 2(e-h)). KEGG pathway analysis identified 272 signaling pathways that were significantly enriched, including 66 associated with the upregulated DEGs and 206 associated with the downregulated DEGs. DEGs were used to analyze and map the KEGG pathways(Figure 3(a)). From the KEGG pathway map, we found that IL-17 signaling pathway, Chemokine signaling pathway, ECM-receptor interaction, as well as Cytokine-cytokine receptor interaction and Influenza A were the top five enriched pathway. In addition, through GSEA, we found that IL-17_SIGNAL_PATHWAY were significantly enriched pathways (Figure 3(e)).

### 3.3 PPI network construction and hub gene identification

The STRING database was used to construct a PPI network of the DEGs, and the results revealed 55 nodes and 173edges. The top 10 hub genes associated with PI were Fn1, Hras, Mapk14, Aurkb, Gnaq, Cdc42, Cxc15, Sgol1, Cenpc1 and Rac1 (Figure 3(c-d), Table 2).

**Table 2.** Degree of top 10 hub genes.

Rank	Gene ID	Gene name	Description	Degree
1	ENSRNOG00000014288	Fn1	fibronectin 1	32
2	ENSRNOG00000016611	Hras	HRas proto-oncogene, GTPase	30
2	ENSRNOG00000000513	Mapk14	mitogen activated protein kinase 14	30
4	ENSRNOG00000005659	Aurkb	aurora kinase B	24
4	ENSRNOG00000014183	Gnaq	G protein subunit alpha q	24
4	ENSRNOG00000013536	Cdc42	cell division cycle 42	24
7	ENSRNOG00000020311	Cxcl5	C-X-C motif chemokine ligand 5	22
7	ENSRNOT00000065721	Sgol1	shugoshin 1	22
7	ENSRNOT00000031074	Cenpc1	Centromere protein C 1	22
7	ENSRNOG00000001068	Rac1	Rac family small GTPase 1	22

### 3.4 Validation by qPCR

The top upregulated DEG (IL-17) from RNA-seq analysis was verified by qPCR, which also demonstrated significantly increased expression of the cytokine in the Sev group, as compared with the WT group (Figure 3(f)).

### 3.5 IL-17A may be an important factor in sevoflurane-induced cognitive impairment in neonatal mice

To reveal the potential mechanism of Repeated sevoflurane exposure induced cognitive impairment, we perfromed RNA-seq analysis between between IL-17A^−/−^ and IL-17A^−/−^ + Sev mice, the results showed L-17A related gene such as Ccl20, Cxcl6, Tnn and Slc4a1 were not enriched (Figure 2(b,d)) and IL-17A signaling pathway were not enriched in KEGG analysis either (Figure 3(b)). These data suggest that IL-17A signaling pathway is an important factor in sevoflurane-induced neurocognitive impairment.

### 3.6 Repeated sevoflurane exposure causes cognitive impairment in neonatal mice that can be ameliorated by IL-17A deletion

**Figure 4.**
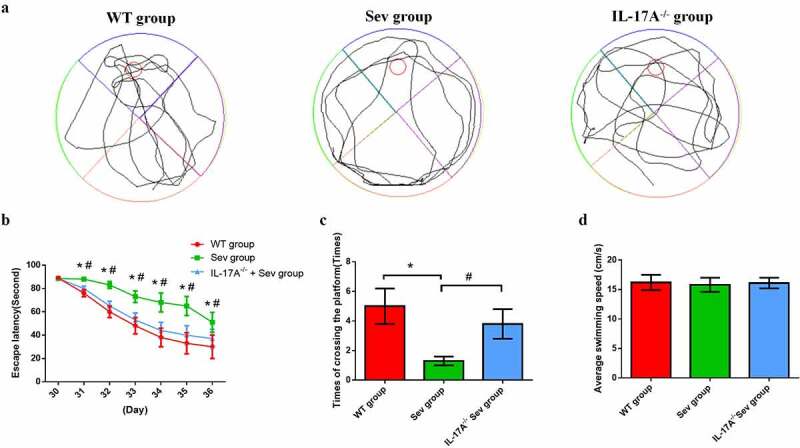
IL-17A deletion ameliorates learning and memory impairment induced by repeated sevoflurane exposure in neonatal mice. (a) Swimming trajectory in spatial exploration experiment. (b) Average escape latency. (c) Times of crossings of the platform area. (d) Average swimming speed in the MWM test. Data are expressed as mean ± SD (*n* = 30 for each group). Compared with the WT group, *P < 0.05; compared with the Sev group, ^#^*P* < 0.05.

The MWM test was used to assess changes in the cognitive ability of neonatal mice. The navigation test revealed no difference in escape latency among the three groups on P30. However, between P31 and P36, the Sev group, as compared with the WT group, exhibited a longer escape latency, which was remarkably reduced by IL-17A deletion (Figure 4(b)). This suggested that multiple sevoflurane exposures cause damage to long term-spatial learning, which could be rescued by IL-17A deletion. The swimming speed analysis showed no significant difference among the three groups (Figure 4(d)). The probe trial demonstrated significant improvement in the IL-17A^−/−^ + Sev group (Figure 4(a,c)) with respect to the decreases in the number of platform crossings as well as time spent in the target quadrant in the Sev group, indicating the potential attenuation of long-term memory impairments after multiple sevoflurane exposures by IL-17A deletion.

### 3.7 IL-17A deletion ameliorates pathological changes after repeated sevoflurane exposure in the hippocampal CA regions of neonatal mice

**Figure 5.**
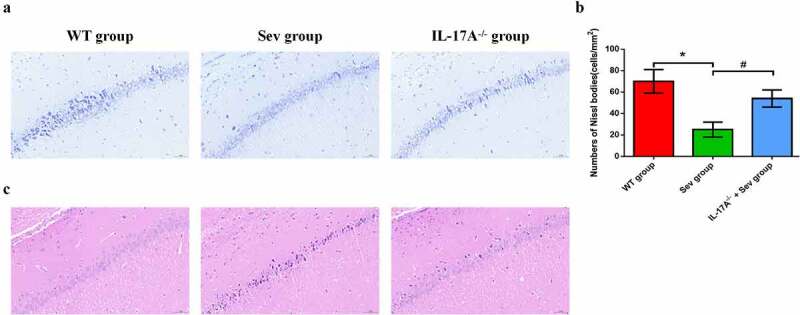
IL-17A deletion ameliorates neuronal injury after repeated sevoflurane exposure in neonatal mice. (a) Representative photomicrographs of Nissl staining in the hippocampal CA1 region; scale bar = 10 μm. (b) Quantification of the number of Nissl bodies using Nissl staining in the hippocampal CA1 region. (c) Representative images of histopathological changes in the hippocampal CA1 of neonatal mice. Data are expressed as mean ± SD (*n* = 5 for each group). Compared with the WT group, *P < 0.05; compared with the Sev group, ^#^*P* < 0.05.

Neuronal counts were assessed by Nissl staining. The histological analysis revealed that, in WT mice, the neurons were aligned and the structure of the CA1 region was normal in the WT group, but that, in comparison, neurons were disorganized after repeated sevoflurane exposure in the Sev group (Figure 5(a)). IL-17A deletion remarkably restored the normal alignment and structure in the CA1 area in the IL-17A^−/−^ + Sev group. Moreover, Nissl bodies were notaly decreased in the Sev group, as compared with the WT group (*P* < 0.01). Notably, IL-17A deletion increased the neuronal counts in the IL-17A^−/−^ + Sev group compared with the Sev group (*P* < 0.01) (Figure 5(b)). As shown in Figure 5(c), in the WT group, the hippocampus appeared fully intact, with hippocampal pyramidal cells showing a normal morphology, with round and pale-stained nuclei and an evenly stained cytoplasm. The hippocampus in the Sev group showed marked disordered neuronal arrangement and acidophilic changes. These pathological changes in the Sev group were, in comparison, alleviated in the IL-17A^−/−^ + Sev group.

### 3.8 Repeated sevoflurane exposure in neonatal mice increases inflammatory factor levels, which can be reduced by IL-17A deletion

**Figure 6.**
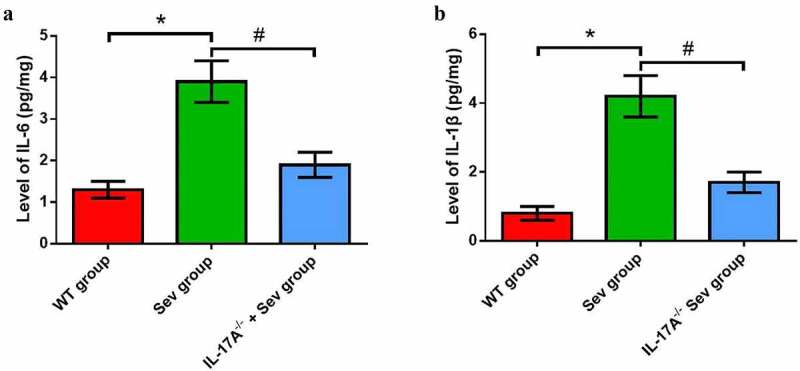
IL-17A deletion diminishes the increase in inflammatory factor levels induced by repeated exposure to sevoflurane in neonatal mice. The levels of hippocampal IL-6 (a) and IL-1β (b). Data are presented as mean ± SD (*n* = 5 for each group). Compared with the WT group, *P < 0.05; compared with the Sev group, ^#^*P* < 0.05.

The levels of inflammatory factors, including IL-1β and IL-6, were substantially increased in the hippocampus of neonatal rats in the Sev group as compared with the WT group. Mice in the IL-17A^−/−^ + Sev group showed reduced IL-1β and IL-6 levels compared with mice in the Sev group (Figure 6(a-b), all *P* < 0.05).

### 3.9 Repeated sevoflurane exposure activates the NF-κB signaling pathway, while IL-17A deletion suppresses this change

**Figure 7.**
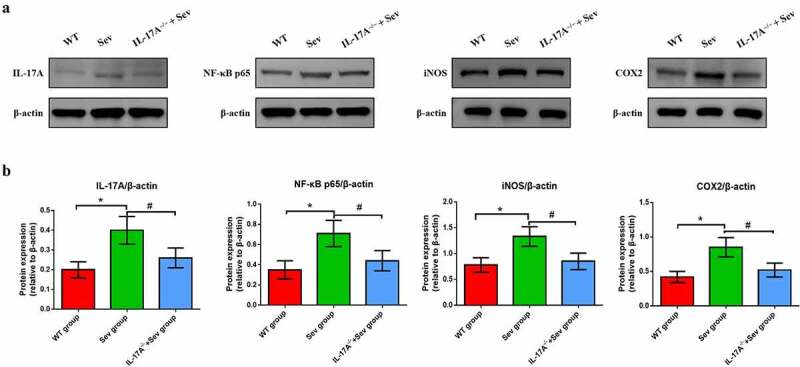
IL-17A deletion ameliorates activation of repeated sevoflurane exposure-induced NF-κB signaling pathway in neonatal mice. (a) Representative western blot of IL-17A, NF-κB p65, iNOS and COX-2. (b) Representative histogram of the relative expression of IL-17A, NF-κB p65, iNOS and COX-2. Data are expressed as mean ± SD (*n* = 10 for each group). Compared with the WT group, *P < 0.05; compared with the Sev group, ^#^*P* < 0.05.

To ascertain the mechanisms by which IL-17A deletion ameliorates cognitive impairment caused by repeat sevoflurane exposure in neonatal mice, we used western blot analysis to examine the changes in the protein levels of IL-17A, NF-κB p65, COX-2 and iNOS. The analysis showed that the IL-17A and NF-κB p65, as well as iNOS and COX-2 were markedly upregulated in the hippocampus of neonatal mice in the Sev group. Notably, neonatal mice in the IL-17A^−/−^ + Sev group showed decreased expression levels of NF-κB p65, IL-17A, iNOS and COX-2 as compared with the Sev group (all *P* < 0.05; Figure 7(a, b)).

### 3.10 IL-17A deletion diminishes the upregulation of NF-κB p65 in hippocampal neurons induced by repeated exposure to sevoflurane in neonatal mice

**Figure 8.**
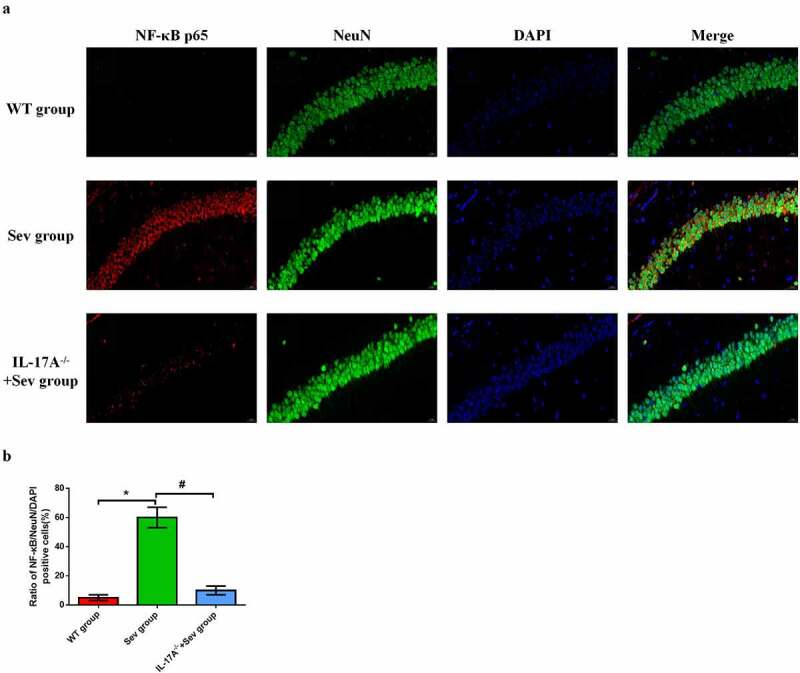
IL-17A deletion reduces the upregulation of NF-κB p65 in repeated sevoflurane exposure-induced hippocampal neuronal cells in neonatal mice. (a) Representative photomicrographs of NF-κB p65/NeuN/DAPI staining (NF-κB p65: red; NeuN: green; DAPI: blue); scale bar = 10 μm. (b) Percentages of NF-κB p65/NeuN/DAPI positive cells. Data are expressed as mean ± SD. Compared with the WT group, *P < 0.05; compared with the Sev group, ^#^*P* < 0.05.

To further elucidate the neuroprotective effect of IL-17A deletion in hippocampal neurons in neonatal mice, immunofluorescence was employed to evaluate the expression of NF-κB p65 in neurons. As shown in Figure 6, NF-κB p65-positive neurons were remarkably higher in neonatal mice exposed to sevoflurane than in those exposed to the carrier gas. IL-17A deletion substantially reduced NF-κB p65 expression in the IL-17A^−/−^ + Sev group compared with the Sev group (all *P* < 0.05, Figure 8(a, b)). These changes are consistent with those seen by western blot analysis.

## 4. Discussion

Studies have shown that repeated exposure of the developing brain to sevoflurane can give rise to neurotoxicity and cognitive impairment in the mature stage; however, the underlying mechanisms are unclear. The current study provides new mechanistic insight into how repeated exposure to sevoflurane in neonatal mice induces long-term cognitive impairment. In hippocampal tissues, a total of 131 DEGs were identifed, and the IL-17 signaling pathway was found to be the most significantly enriched from RNA-seq analysis. IL-17A deletion ameliorated long-term cognitive impairment induced by multiple sevoflurane exposure in neonatal mice, inhibited the activation of the NF-κB signaling pathway, and led to alleviated neuroinflammation.

Learning and memory are the most important functions of the brain, one of the advanced nervous functions and an important indicator of human intellectual development [30]. For many years, people have done a lot of research on the localization of learning and memory in the brain, and they believe that learning and memory processes depend on the participation of multiple brain regions, such as the cortex, hippocampus and amygdala [31]. Hippocampus is an important brain area related to learning and memory. It is not only closely related to memory function, but also related to cognitive function and location navigation. Recent studies have confirmed the hippocampus exerts a particularly critical effect in the consolidation of memory and the formation of spatial memory [32].

The developing brain (up to 3 weeks in rodents) is more vulnerable to the influence of external factors, such as anesthesia [33,34]. In the pretest, we found that there was a significant difference in the long-term cognitive function of mice given a single or multiple (3 times) inhalation of a subclinical dose of sevoflurane. A single treatment (2 h) did not affect their long-term learning and memory function, but repeated treatment could damage their long-term cognitive function. Shen found that exposure to 3% sevoflurane for 2 hours daily for 3 consecutive days did not cause cognitive impairment in adult mice (60 days of age), compared with neonatal mice (6 days of age) [35]. This finding has been confirmed by clinical trials – early anesthesia/surgery exposure may be responsible for an increased risk of cognitive impairment in children [36]. For 6-day-old neonatal mice, 3% sevoflurane is about 0.6MAC, which is lower than the clinical anesthesia concentration. This is mainly because the experiment only examines the effect of anesthesia on the neural development of neonatal mice without any surgical trauma stimulation. If 1MAC sevoflurane anesthesia is used, it may lead to excessive anesthesia, easily induce inhibition of respiration and circulation, interfere with the accuracy of the experimental results, and may increase the mortality of neonatal mice. The duration of each anesthesia was 2 h, which was also to reduce the interference of sevoflurane on the physiological function of newborn mice. According to the method of Shen, we conducted the MWM test at 30–36 days after birth for evaluation of the long-term impact of sevoflurane on cognitive function [35]. The MWM is a classical approach to evaluation of the spatial cognitive function of rodents. It is divided into a positional navigation experiment and a spatial exploration experiment. The positional navigation experiment reveals the spatial learning ability of animals, and the spatial exploration experiment reflects the spatial memory ability [37]. A shorter escape latency and increased crossings of the original platform area are indicative of stronger learning and memory abilities. The escape latency in this study was prolonged at P32–36 days, and the number of platform crossings was lower on P36 in the Sev group. Notably, these changes were reduced in the IL-17A^−/−^ + Sev group. These findings reveal that multiple exposure to sevoflurane can produce long-term cognitive impairment, and that IL-17A knockout can improve the cognitive impairment, consistent with Shen’s study [35].

We performed RNA-seq analysis to identify DEGs associated with sevoflurane-induced neurocognitive impairment in neonatal mice. RNA-seq, also known as transcriptome sequencing technology, is a method for identifying all RNAs transcribed by a specific cell, tissue or individual at a specific time and/or state using high-throughput sequencing technology [38,39]. We found here that the immune response was enriched by GO analysis, and that the IL-17 signaling pathway was significantly enriched by KEGG analysis, consistent with Yang’s study [22]. Meanwhile, we performed another RNA-seq analysis between IL-17A^−/−^ and IL-17A^−/−^ + Sev mice, the results showed IL-17A related gene and pathway cannot be enriched, indicating that IL-17A is an important factor in sevoflurane-induced neurocognitive impairment.

A healthy immune response is essential for appropriate wound healing and repair following tissue damage, and for fighting infection without damaging the host’s own cells or tissues. However, an excessive inflammatory response is of great harm, which may lead to serious tissue damage and even death. Sevoflurane is the most commonly employed inhaled anesthetic in pediatric anesthesia [40], and repeated sevoflurane anesthesia can cause neuroinflammatory reactions in young mice and affect long-term cognitive functions [41]. Repeated sevoflurane exposure can destroy the blood–brain barrier, causing the release of inflammatory factors such as IL-1β and IL-6 [42]. Zhang showed that sevoflurane increases IL-6 and IL-1β levels via activation of the NF-κB pathway [43]. In this study, we found that the expression levels of IL-1β, IL-6 and pathological injury of hippocampus were increased significantly in the Sev group, and that IL-17A deletion could suppress these changes, indicating that IL-17A deletion ameliorates multiple sevoflurane exposure-induced neuroinflammation.

The IL-17 family comprises six members (IL-17A–F), among which IL-17A is the most well-studied [17]. Th17 cells typically secrete IL-17A, and other cells such as γδ T cells, CD8^+^ T cells as well as neutrophils and eosinophils also secrete IL-17A. IL-17 binds to IL-17 receptors expressed on epithelial cells as well as on endothelial cells and fibroblasts, resulting in the release of cytokines, including IL-1, IL-6, IL-8, IL-23 and TNF-α, as well as other chemokines to initiate an inflammatory cascade. IL-17A is therefore considered a powerful proinflammatory factor [15]. NF-κB is a eukaryotic transcription factors consisting of five subunits – p65, Re1B, c-Re1, NF-κB1 and NF-κB2. NF-κB is able to regulate the expression of cytokines and modulate the immune system [44]. Several studies [43,45] and our previous report [46] suggested that the NF-κB signaling pathway plays a pivotal role in the sevoflurane-induced neuroinflammatory response. iNOS is a pro-inflammatory enzyme that produces NO. iNOS can be activated by various stimuli to produce NO, which in excessive amounts can induce an inflammatory response. COX-2 is the main participant in the inflammatory response in peripheral tissues. The expression of COX-2 in the brain is closely associated with the level of the neuroinflammatory response. COX-2 is involved in the pathogenesis of neurodegenerative diseases. NF-κB is implicated in regulating the expression of iNOS and COX-2. Thus, blocking NF-κB activation can inhibit iNOS and COX-2 expression [47]. In this study, we found that the expression levels of IL-17A, NF-κB p65, COX-2 and iNOS were remarkably increased in the Sev group, and that IL-17A deletion could suppress these changes, indicating that IL-17A deletion ameliorates multiple sevoflurane exposure-induced neurocognitive impairment by inhibiting the NF-κB signaling pathway.

There are some limitations in this study. First, we only examined the changes in cognitive function and neuroinflammation-related indicators after multiple sevoflurane exposure in neonatal mice from P30 to P36. The changes in cognitive function on P60 and even beyond need to be further explored. Second, we did not study the impact of anesthesia on other domains of cognitive function, such as executive function. On the contrary, we focused on learning and memory, as they are the major domains to undergo decline.

## 5. Conclusion

Our findings reveal that repeated sevoflurane exposure induces significant long-term cognitive impairment, and that IL-17A deletion can prevent these deficits in neonatal mice. The underlying neuroprotective mechanisms may involve inhibition of the NF-κB signaling pathway and suppression of the neuroinflammatory reaction.

## Supplementary Material

Supplemental Material

## Data Availability

The datasets generated and/or analysed during the current study are available from the corresponding author on reasonable request.

## References

[1] Warner DO, Zaccariello MJ, Katusic SK, et al. Neuropsychological and behavioral outcomes after exposure of young children to procedures requiring general anesthesia: the mayo anesthesia safety in kids (MASK) study. Anesthesiology. 2018;129(1):89–105. 29672337 10.1097/ALN.0000000000002232 PMC6008202

[2] Sevoflurane. In: Drugs and lactation database (LactMed). Bethesda (MD): National Library of Medicine (US); 2020.

[3] Brioni JD, Varughese S, Ahmed R, et al. A clinical review of inhalation anesthesia with sevoflurane: from early research to emerging topics. J Anesth. 2017;31:764–778. 28585095 10.1007/s00540-017-2375-6 PMC5640726

[4] Fan XY, Shi G, Zhao P. Neonatal sevoflurane exposure impairs learning and memory by the hypermethylation of hippocampal synaptic genes. Mol Neurobiol. 2021;58:895–904. 33052583 10.1007/s12035-020-02161-4

[5] Wilder RT, Flick RP, Sprung J, et al. Early exposure to anesthesia and learning disabilities in a population-based birth cohort. Anesthesiology. 2009;110:796–804. 19293700 10.1097/01.anes.0000344728.34332.5d PMC2729550

[6] Huang X, Ying J, Yang D, et al. The mechanisms of sevoflurane-induced neuroinflammation. Front Aging Neurosci. 2021;13:717745. 34421578 10.3389/fnagi.2021.717745 PMC8375153

[7] Wang CM, Chen WC, Zhang Y, et al. Update on the mechanism and treatment of sevoflurane-induced postoperative cognitive dysfunction. Front Aging Neurosci. 2021;13:702231. 34305576 10.3389/fnagi.2021.702231 PMC8296910

[8] Chai D, Cheng Y, Jiang H. Fundamentals of fetal toxicity relevant to sevoflurane exposures during pregnancy. Int J Dev Neurosci. 2019;72:31–35. 30448514 10.1016/j.ijdevneu.2018.11.001

[9] Dang DD, Saiyin H, Yu Q, et al. Effects of sevoflurane preconditioning on microglia/macrophage dynamics and phagocytosis profile against cerebral ischemia in rats. CNS Neurosci Ther. 2018;24:564–571. 29427321 10.1111/cns.12823 PMC6490012

[10] Acharya NK, Goldwaser EL, Forsberg MM, et al. Sevoflurane and isoflurane induce structural changes in brain vascular endothelial cells and increase blood−brain barrier permeability: possible link to postoperative delirium and cognitive decline. Brain Res. 2015;1620:29–41. 25960348 10.1016/j.brainres.2015.04.054

[11] Han C, Zhang Z, Guo N, et al. Effects of sevoflurane inhalation anesthesia on the intestinal microbiome in mice. Front Cell Infect Microbiol. 2021;11:633527. 33816336 10.3389/fcimb.2021.633527 PMC8012717

[12] Chen R, Zhang T, Kuang L, et al. Cholinergic synaptic transmissions were altered after single sevoflurane exposure in Drosophila pupa. Biomed Res Int. 2015;2015:485709. 25705662 10.1155/2015/485709 PMC4331166

[13] Wang F, Li C, Shao J, et al. Sevoflurane induces inflammation of microglia in hippocampus of neonatal rats by inhibiting Wnt/β-Catenin/CaMKIV pathway. J Pharmacol Sci. 2021;146(2):105–115. 33941321 10.1016/j.jphs.2021.02.004

[14] Yin J, Zhao X, Wang L, et al. Sevoflurane-induced inflammation development: involvement of cholinergic anti-inflammatory pathway. Behav Pharmacol. 2019;30(8):730–737. 31625977 10.1097/FBP.0000000000000507

[15] Brembilla NC, Senra L, Boehncke W-H. The IL-17 Family Of Cytokines In Psoriasis: IL-17A and beyond. Front Immunol. 2018;9:1682. 30127781 10.3389/fimmu.2018.01682 PMC6088173

[16] Rouvier E, Luciani MF, Mattéi MG, et al. CTLA-8, cloned from an activated T cell, bearing AU-rich messenger RNA instability sequences, and homologous to a herpesvirus saimiri gene. J Iimmunol. 1993;150:5445–5456. 8390535

[17] McGeachy MJ, Cua DJ, Gaffen SL. The IL-17 family of cytokines in health and disease. Immunity. 2019;50(4):892–906. 30995505 10.1016/j.immuni.2019.03.021 PMC6474359

[18] Ness-Schwickerath KJ, Morita CT. Regulation and function of IL-17A- and IL-22-producing γδ T cells. Cell Mol Life Sci. 2011;68:2371–2390. 21573786 10.1007/s00018-011-0700-z PMC3152582

[19] Cristiano C, Volpicelli F, Lippiello P, et al. Neutralization of IL-17 rescues amyloid-β-induced neuroinflammation and memory impairment. Br J Pharmacol. 2019;176:3544–3557. 30673121 10.1111/bph.14586 PMC6715610

[20] Shabab T, Khanabdali R, Moghadamtousi SZ, et al. Neuroinflammation pathways: a general review. Int J Neurosci. 2017;127:624–633. 27412492 10.1080/00207454.2016.1212854

[21] Tóbon-Velasco JC, Cuevas E, Torres-Ramos MA. Receptor for AGEs (RAGE) as mediator of NF-kB pathway activation in neuroinflammation and oxidative stress. CNS Neurol Disord Drug Targets. 2014;13:1615–1626. 25106630 10.2174/1871527313666140806144831

[22] Yang ZY, Yuan CX. IL-17A promotes the neuroinflammation and cognitive function in sevoflurane anesthetized aged rats via activation of NF-κB signaling pathway. BMC Anesthesiol. 2018;18:147. 30342469 10.1186/s12871-018-0607-4 PMC6195755

[23] Yu Y, Yang Y, Tan H, et al. Tau contributes to sevoflurane-induced neurocognitive impairment in neonatal mice. Anesthesiology. 2020;133:595–610. 32701572 10.1097/ALN.0000000000003452 PMC7429299

[24] Shiroguchi K, Jia TZ, Sims PA, et al. (2012) Digital RNA sequencing minimizes sequence-dependent bias and amplification noise with optimized single-molecule barcodes. Proc Natl Acad Sci U.S.A 109:1347–1352 22232676 10.1073/pnas.1118018109 PMC3268301

[25] Gu Z, Eils R, Schlesner M. Complex heatmaps reveal patterns and correlations in multidimensional genomic data. Bioinformatics. 2016;32:2847–2849. 27207943 10.1093/bioinformatics/btw313

[26] Yu G, Wang LG, Han Y, et al. Cluster profiler: an R package for comparing biological themes among gene clusters. OMICS. 2012;16:284–287. 22455463 10.1089/omi.2011.0118 PMC3339379

[27] Szklarczyk D, Franceschini A, Wyder S, et al. STRING v10: protein-protein interaction networks, integrated over the tree of life. Nucleic Acids Res. 2015;43:D447–452. 25352553 10.1093/nar/gku1003 PMC4383874

[28] Chin CH, Chen SH, Wu HH, et al. cytoHubba: identifying hub objects and sub-networks from complex interactome. BMC Syst Biol. 2014;8(Suppl 4):S11. 25521941 10.1186/1752-0509-8-S4-S11 PMC4290687

[29] Carriel V, Campos A, Alaminos M, et al. Staining methods for normal and regenerative myelin in the nervous system. Methods Mol Biol. 2017;1560:207–218. 28155156 10.1007/978-1-4939-6788-9_15

[30] Cassilhas RC, Tufik S, de Mello MT. Physical exercise, neuroplasticity, spatial learning and memory. Cell Mol Life Sci. 2016;73:975–983. 26646070 10.1007/s00018-015-2102-0 PMC11108521

[31] Yavas E, Gonzalez S, Fanselow MS. Interactions between the hippocampus, prefrontal cortex, and amygdala support complex learning and memory. F1000Res. 2019;8:1292. 10.12688/f1000research.19317.1 PMC6676505 31448084

[32] Bettio LEB, Rajendran L, Gil-Mohapel J. The effects of aging in the hippocampus and cognitive decline. Neurosci Biobehav Rev. 2017;79:66–86. 28476525 10.1016/j.neubiorev.2017.04.030

[33] Rice D, Barone S Jr. Critical periods of vulnerability for the developing nervous system: evidence from humans and animal models. Environ Health Perspect. 2000;108(Suppl 3):511–533. 10852851 10.1289/ehp.00108s3511 PMC1637807

[34] Stratmann G, Sall JW, May LD, et al. Isoflurane differentially affects neurogenesis and long-term neurocognitive function in 60-day-old and 7-day-old rats. Anesthesiology. 2009;110:834–848. 19293705 10.1097/ALN.0b013e31819c463d

[35] Shen X, Dong Y, Xu Z, et al. Selective anesthesia-induced neuroinflammation in developing mouse brain and cognitive impairment. Anesthesiology. 2013;118:502–515. 23314110 10.1097/ALN.0b013e3182834d77 PMC3580002

[36] Flick RP, Katusic SK, Colligan RC, et al. Cognitive and behavioral outcomes after early exposure to anesthesia and surgery. Pediatrics. 2011;128:e1053–1061. 21969289 10.1542/peds.2011-0351 PMC3307194

[37] Ravichandran VA, Kim M, Han SK, et al. Stachys sieboldii extract supplementation attenuates memory deficits by modulating BDNF-CREB and its downstream molecules, in animal models of memory impairment. Nutrients. 2018;10;7:917 . 10.3390/nu10070917 PMC6073797 30018265

[38] Hrdlickova R, Toloue M, Tian B. RNA-seq methods for transcriptome analysis. Wiley Interdiscip Rev RNA. 2017;8:10. 10.1002/wrna.1364 PMC5717752 27198714

[39] Wang Z, Gerstein M, Snyder M. RNA-Seq: a revolutionary tool for transcriptomics. Nat Rev Genet. 2009;10:57–63. 19015660 10.1038/nrg2484 PMC2949280

[40] Hobbhahn J, Funk W. Sevoflurane in pediatric anesthesia. Anaesthesist. 1996;45(Suppl 1):S22–27. 8775099

[41] Li T, Huang Z, Wang X, et al. Role of the GABAA receptors in the long-term cognitive impairments caused by neonatal sevoflurane exposure. Rev Neurosci. 2019;30:869–879. 31145696 10.1515/revneuro-2019-0003

[42] Zheng JW, Meng B, Li XY, et al. NF-κB/P65 signaling pathway: a potential therapeutic target in postoperative cognitive dysfunction after sevoflurane anesthesia. Eur Rev Med Pharmacol Sci. 2017;21:394–407. 28165545

[43] Zhang L, Zhang J, Yang L, et al. Isoflurane and sevoflurane increase interleukin-6 levels through the nuclear factor-kappa B pathway in neuroglioma cells. Br J Anaesth. 2013;110 Suppl 1:i82–91. 23604542 10.1093/bja/aet115 PMC3667345

[44] Mitchell JP, Carmody RJ. NF-κB and the Transcriptional Control of Inflammation. Int Rev Cell Mol Biol. 2018;335:41–84. 29305014 10.1016/bs.ircmb.2017.07.007

[45] Wang Q, Zhao Y, Sun M, et al. 2-Deoxy-d-glucose attenuates sevoflurane-induced neuroinflammation through nuclear factor-kappa B pathway in vitro. Toxicol Vitro. 2014;28:1183–1189. 10.1016/j.tiv.2014.05.006 24907647

[46] Zhao Z, Ma L, Li Y, et al. MiR-124 protects against cognitive dysfunction induced by sevoflurane anesthesia in vivo and in vitro through targeting calpain small subunit 1 via NF-κB signaling pathway. Adv Clin Exp Med. 2021;30:701–709. 34118141 10.17219/acem/134740

[47] Surh YJ, Chun KS, Cha HH, et al. Molecular mechanisms underlying chemopreventive activities of anti-inflammatory phytochemicals: down-regulation of COX-2 and iNOS through suppression of NF-kappa B activation. Mutat Res. 2001;480-481:243–268. 11506818 10.1016/s0027-5107(01)00183-x

