# Supplementary material for: IL-17A deletion reduces sevoflurane-induced neurocognitive impairment in neonatal mice by inhibiting NF-κB signaling pathway
Source: Bioengineered. 2022 Jun 26;13(6):14562–77. doi: 10.1080/21655979.2022.2090608 (PMC9342424; doi:10.1080/21655979.2022.2090608)

**IL17A**

5’-GCC TCC AAG TCT AGC TTT GCT-3’ Mutant Forward

5’-CCT AAG GAG GTC CGG AAT GT-3’ Mutant Reverse

5’-GCA TCC CAG ACC AGC ATT AG-3’ Wild type Forward

5’-CCA GCT GTC ATC CAG ACA AG-3’ Wild type Reverse

Mutant = ~450 bp

Heterozygote = ~450 bp and 201 bp

Wild type = 201 bp


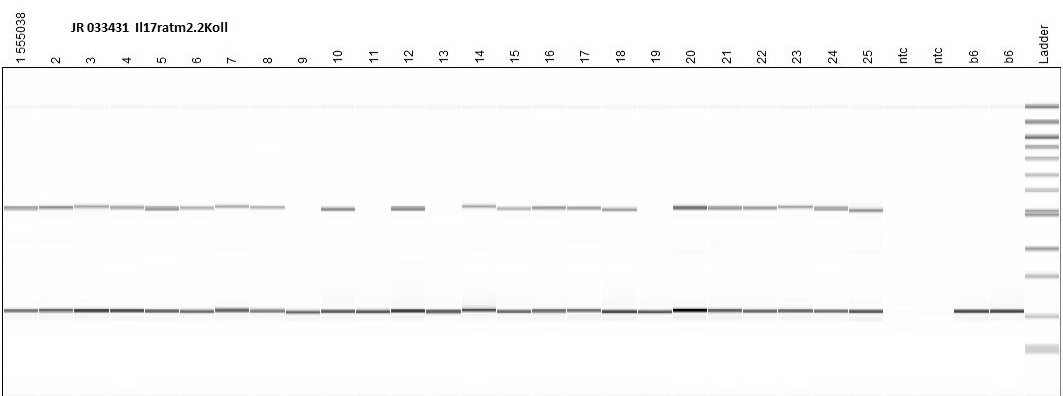

Supplement: Supplemental Material [file KBIE_A_2090608_SM3564.zip › supplementary/Aprimeridentificationsequence.docx]
